# Beta Band Corticomuscular Drive Reflects Muscle Coordination Strategies

**DOI:** 10.3389/fncom.2017.00017

**Published:** 2017-04-04

**Authors:** Alexander Reyes, Christopher M. Laine, Jason J. Kutch, Francisco J. Valero-Cuevas

**Affiliations:** ^1^Brain-Body Dynamics Lab, Department of Biomedical Engineering, University of Southern CaliforniaLos Angeles, CA, USA; ^2^Applied Mathematical Physiology Lab, Division of Biokinesiology and Physical Therapy, University of Southern CaliforniaLos Angeles, CA, USA

**Keywords:** coherence, dexterity, beta-band, neuromuscular, synergy, EMG, EEG

## Abstract

During force production, hand muscle activity is known to be coherent with activity in primary motor cortex, specifically in the beta-band (15–30 Hz) frequency range. It is not clear, however, if this coherence reflects the control strategy selected by the nervous system for a given task, or if it instead reflects an intrinsic property of cortico-spinal communication. Here, we measured corticomuscular and intermuscular coherence between muscles of index finger and thumb while a two-finger pinch grip of identical net force was applied to objects which were either stable (allowing synergistic activation of finger muscles) or unstable (requiring individuated finger control). We found that beta-band corticomuscular coherence with the first *dorsal interosseous* (FDI) and *abductor pollicis brevis* (APB) muscles, as well as their beta-band coherence with each other, was significantly reduced when individuated control of the thumb and index finger was required. We interpret these findings to show that beta-band coherence is reflective of a synergistic control strategy in which the cortex binds task-related motor neurons into functional units.

## Introduction

Both corticomuscular and intermuscular synchronization, as quantified by coherence analysis (Rosenberg et al., 1989; Farmer et al., 1993; Conway et al., 1995), provide an important means of understanding the cortical drive to muscles (Conway et al., 1995; Baker et al., 1997; Brown, 2000; Boonstra et al., 2009a). Corticomotor drive contains a 15–30 Hz (beta-band) oscillatory component (Murthy and Fetz, 1992, 1996a,b; Sanes and Donoghue, 1993; Stancák and Pfurtscheller, 1996; Donoghue et al., 1998; Mima and Hallett, 1999; Lebedev and Wise, 2000; Witham et al., 2010), which entrains targeted motor neurons (Farmer et al., 1993, 1997; Mima and Hallett, 1999) and leads to synchronization between cortical and muscular activities in that frequency range (Murthy and Fetz, 1992; Conway et al., 1995; Baker et al., 1997; Salenius et al., 1997). Functionally-related muscles also share a common intermuscular beta-band input (Kilner et al., 1999; Boonstra and Breakspear, 2012; Boonstra, 2013), which is widely accepted as cortical in origin (Brown et al., 1999), as the motor cortex is the only well-established source for such beta-band drive.

Although beta-band cortical drive has received a great deal of attention, its functional significance for motor control remains unclear. Currently it is suggested that oscillations in this range functions to support a constant motor state (Kilner et al., 2000; Pogosyan et al., 2009; Engel and Fries, 2010). This is consistent with the observation that, during the production of a constant force, beta-band coherence is strengthened with continued sensory feedback and minimal voluntary movement (Gilbertson et al., 2005; Androulidakis et al., 2006, 2007; Lalo et al., 2007; Engel and Fries, 2010; Aumann and Prut, 2015). While the magnitude of beta-band corticomuscular coherence does correlate with force(Conway et al., 1995; Baker et al., 1997; Kilner et al., 1999, 2000, 2004; Baker, 2007; Kristeva et al., 2007; Witte et al., 2007), it disappears during movement (Baker et al., 1997; Kilner et al., 1999, 2000, 2004; Brown, 2000; Feige et al., 2000) and imagined movements (De Lange et al., 2008), and there is even evidence that the signal is not entirely feed-forward (Fisher et al., 2002; Baker et al., 2006; Witham et al., 2011). Such findings raise important questions as to the functional role of beta-band drive to muscles.

Numerous studies have investigated low force (<5 N) precision pinch paradigms to characterize cortico-spinal interactions through the use of corticomuscular coherence (CMC) (Muir and Lemon, 1983; Lemon and Mantel, 1989; Lemon et al., 1995, 1998; Baker et al., 2003). Findings have revealed that beta-band CMC is modulated by digit displacement (Riddle and Baker, 2006), object compliance (Kilner et al., 2000), and similar studies suggest a dependence upon the history and time course of muscle contraction (Chakarov et al., 2009; Omlor et al., 2011; Nazarpour et al., 2012). In nearly all cases, findings of decreased beta-band CMC can be interpreted as reflecting either (1) a departure from steady-state control of a particular muscle, or (2) the cortical “unbinding” of muscles when individuated, rather than synergistic, activation is called for. Given that beta-band cortical activity has been suggested as a “binding” signal for many years (Gray, 1994; Santello, 2014), and that such binding would naturally favor synergistic rather than individuated control of the fingers (Boonstra et al., 2009b; Danna-Dos Santos et al., 2010; Kattla and Lowery, 2010; Aumann and Prut, 2015), our overall hypothesis was that beta-band corticomotor drive should be reduced or eliminated when the degree of individuated muscle control is increased.

To address these issues, we studied beta-band corticomuscular and inter-muscular coherence (CMC and IMC, respectively) while participants applied low magnitude precision pinch forces to one of two different objects. The first object was a solid wooden dowel. Production of a constant pinch force against a solid object represents a relatively simple task for the nervous system. The second object was a custom-designed spring which buckles when compressed unless prevented from doing so through precise dynamic adjustment of thumb and index fingertip forces. The spring task described herein has been modified from Valero-Cuevas et al. (2003) and described in Dayanidhi et al. (2013b). This task requires the dynamic regulation of thumb and index fingertip force vectors in 3-D to stabilize the spring, which can be modeled as undergoing an instability similar to a subcritical pitchfork bifurcation (Venkadesan et al., 2007). The physical movement of the fingers remains negligible because large movements tend to increase the likelihood of buckling.

If beta-band coherence depends on the generation of a relatively stable pinch force, then coherence should change relatively little across these two tasks. If beta-band coherence is inherently an intermuscular binding signal, then we would expect to see little coherence during compression of the unstable spring, either in terms of CMC or IMC. Our findings support this hypothesis and suggest that the dynamic, mechanical relationships among muscles are likely critical factors shaping the frequency content of corticomotor drive.

## Methods

### Participants

We recruited 15 healthy participants (30.3 ± 4.6 years, 6 females) who were self-reported as right-handed. There were no known prior or current neurological conditions in any of the participants, nor did they report any previous hand injuries or surgeries. This study was carried out in accordance with the recommendations of Institutional Review Board (IRB) at the University of Southern California (USC) with written informed consent from all subjects. All subjects gave written informed consent in accordance with the Declaration of Helsinki. The protocol was approved by the Office for the Protection of Research Subjects at USC.

### Task 1: establishing maximal unstable spring compression force

**Figure 1:**
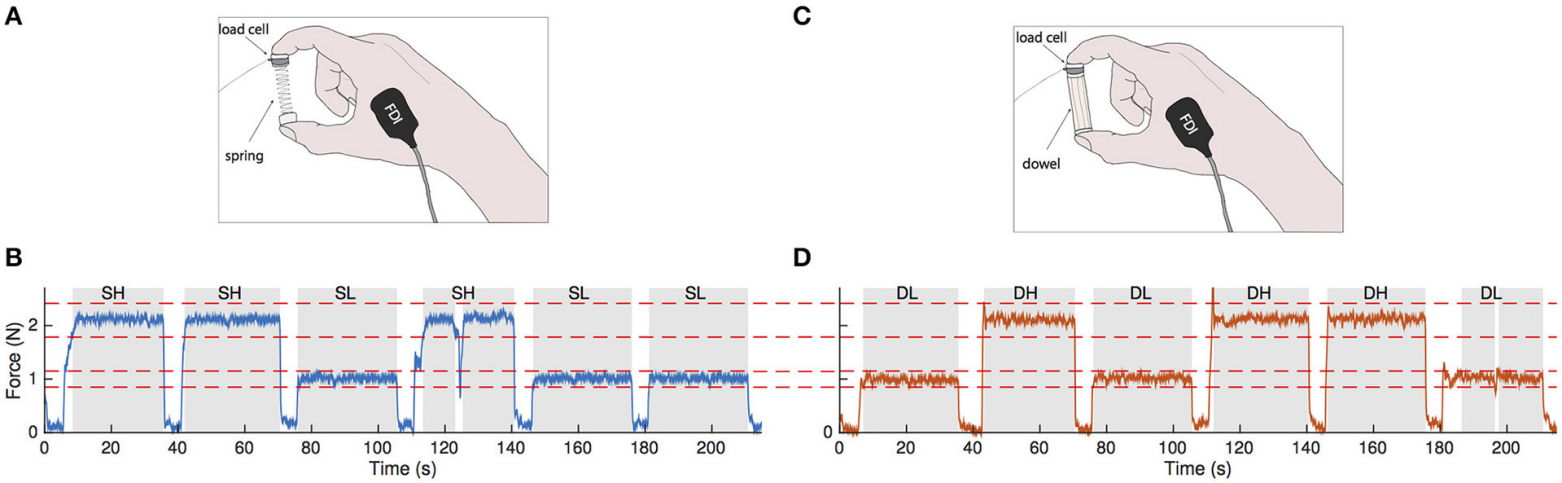
**Visuomotor force tracking paradigm for a representative subject. (A)** A small compliant spring is compressed between the index finger and thumb. Fingertip forces and bipolar surface EMG from the intrinsic hand muscles FDI and APB (not shown) were recorded. **(B)** Force tracking task showing three random presentations during spring compression at forces of 1.0 and 2.1 N. Each force level (high or low) was presented to the subject for 30 s with an inter-stimulus interval of 5 s. The blue trace represents the recorded compression force. Red dotted lines indicate the ±15% tolerance limits relative to target force. Data that are within the tolerance limits for a minimum of 5 s in duration (shaded regions) were used in the coherence analysis. In the fourth hold, the compression force briefly fell out of the tolerance range. **(C)** The visuomotor task is repeated with a stable wooden dowel. **(D)** Force tracking profile with the dowel at the same force levels and tolerance limits as with the spring. Gray areas represent data within tolerance and dowel-low and dowel-high refer to dowel-low and dowel-high, respectively.

The Strength-Dexterity test provides a quantitative measure of hand dexterity by requiring dynamic regulation of endpoint force direction and magnitude to stabilize a slender and compliant spring prone to bucking (Valero-Cuevas et al., 2003). The instability in the spring increases with compression force, and thus the maximal compression force reached is indicative of the greatest instability the neuromuscular system can control. The force required to bring the spring to solid length was approximately 3.7–3.8 N, however the maximal compression force healthy adults can reach is less than 3.0 N (Dayanidhi, 2012). We specifically chose a spring with a low strength requirement (<15% maximal precision pinch force) to focus primarily on cortical drive involved with dexterity demand, rather than strength. The resting length of the spring measured 4.2 cm in length, weighed approximately two grams and had a spring constant of 0.86 N/cm. Custom designed 3-D printed acrylonitrile butadiene styrene (ABS) plastic end caps were glued to both ends of the spring to create flat surfaces on which to attach a force transducer. Additional ABS end caps were attached on top of the sensor for two purposes: (1) to provide a place for subjects to grasp the object and (2) to serve as a thermal barrier to prevent body heat from adding a bias to the temperature-sensitive force transducer. With the addition of the end caps and sensor, the effective resting length of the spring was 5.7 cm (Figure 1A).

In this task, subjects rested their right arm on a table and used their index finger and thumb to compress the spring with their hand resting on the table. During the task, we asked participants to ensure that their 3rd–5th fingers did not assist in the task by tucking them into their palm. They were given four attempts (90 s each) to try to compress the spring as much as possible (Valero-Cuevas et al., 2003; Dayanidhi et al., 2013a,b; Lawrence et al., 2014). The average maximal compression force reached prior to spring buckling was taken as a normalized measure of their dexterous performance. We rounded this maximal value to the nearest tenth of a Newton and defined this as the subject-specific F_max_. We then calculated 40 and 80% of the subject-specific F_max_ for use in the second phase of the study.

### Task 2: visuomotor force tracking

Subjects were seated comfortably in front of a computer providing visual feedback of their precision pinch compression force. They compressed either the same slender spring or a wooden dowel (length = 5.2 cm, diameter = 0.12 cm, Figure 1C) to visually track a series of randomly presented step targets set to 40 and 80% of their F_max_. For three trials for a given object, target force levels were presented at 30 s intervals with 5 s of rest in between (Figures 1B,D). The trial lasted until five repetitions of each force level were presented. During the resting periods, the subjects would hold the object with just enough force to prevent dropping it. Breaks were given in between trials when necessary to prevent fatigue effects. Subjects then repeated the procedure with the other object for the same number of trials and force levels. This two-by-two factorial design yielded four force conditions: spring-low, spring-high, dowel-low, and dowel-high.

### Recordings

#### Force

Normal compression forces were measured by affixing a uni-axial load cell (ELFF-B4-10L, Measurement Specialties, Hampton, VA) with double-sided tape to the index finger side of either the compliant spring or the wooden dowel. The circular load cell measured 0.41 cm in height, 1.27 cm in diameter and aligned perfectly with the diameter of the objects. Signals from the sensor were differentially amplified with a custom designed circuit operating in the 0–5 N range. Data were captured using a USB Data Acquisition (DAQ) system (National Instruments, Austin, TX) sampling at a rate of 2,048 Hz. Prior to data collection, the sensor voltage was converted to Newtons by removing the DC offset and calibrating the load-cell using a four-point linear regression with fixed weights. The offset and gain of the load cell were corrected periodically to ensure accurate force recordings.

#### Electromyography (EMG)

Bipolar surface EMG were collected using a Delsys Bagnoli system (Delsys, Natick, MA) from the first *dorsal interosseous* (FDI) and *abductor pollicis brevis* (APB). Data were filtered between 20 and 450 Hz, amplified by 1000, and then sampled at a 2048 Hz. The reference electrode for the recordings was placed on the olecranon of the right arm. Recording locations were identified by palpating the muscle during force production in the direction of mechanical action for each muscle.

#### Electroencephalography (EEG)

Sixty four channels of EEG were recorded at a sampling rate of 2048 Hz (ANT Neuro, Enschede, The Netherlands). The fixed recording sites were arranged according to the international 10–20 system for scalp electrode placement. We ensured repeatable recordings of cortical areas across subject by taking skull measurements and placing electrode Cz at the cross section of the midway point between the nasion and inion and the midway point between the left and right tragus of the ear. Electrode impedances were kept below 10 k with respect to the reference electrode CPz.

Following digitization, EEG signals were bandpass filtered between 10 and 500 Hz and both EEG and EMG signals were notch filtered at 60 Hz and its harmonics up to 500 Hz using a 4th order Butterworth filter implemented in MATLAB (Mathworks, Natick, MA) and FieldTrip, a software package for EEG and EMG analysis (Delorme and Makeig, 2004; Oostenveld et al., 2011). Subsequently, EMG were rectified to extract group activity of motor units (Halliday et al., 1995; Mima and Hallett, 1999). Data formats collected from two separate systems were synchronized by configuring the NI-DAQ to send a trigger pulse to the EEG and EMG systems via a split BNC cable. Custom scripts were created to read in trigger events and synchronize all data.

### Trial selection

The start of a trial was defined as the time when the on-screen target transitioned from a resting value to either 40 or 80% of F_max_, and its end was defined as the time when the target value returned back to rest. Trial windows were 30 s in duration with a 5 s inter-stimulus interval. Sample traces of the matching paradigm are shown in in Figure 1B for the spring (blue trace) and in Figure 1D for the dowel (orange trace); each showing three randomized presentations of the low and high force levels (note that the force levels are the same across objects). Force data during these steady hold phases were visually examined to determine if the task was performed correctly. Our requirement was that the hold phase must be within a ±15% tolerance of the target force value and be held within this range for at least 5 s. The force profiles meeting these criteria are shown as the gray shaded areas in Figures 1B,D. Force profiles not meeting these criteria were excluded from analysis. The accuracy of the force matching task was analyzed by taking the root mean square error of the steady hold force compared to the target level for each of the four conditions.

Epochs of synchronized EEG and EMG from each condition that satisfied the steady-state criteria were normalized and pooled across conditions and subjects (Amjad et al., 1997). Normalizing each epoch of data gives equal weight to each section and effectively eliminates the possibility of coherence bias which favors sections with high EMG amplitude (Amjad et al., 1997; James et al., 2008; Schoffelen et al., 2011).

### Coherence analysis

Synchronous oscillations between cortical activity and EMG indicate functional connectivity which can be assessed through coherence analysis (Nunez et al., 1997; Mima and Hallett, 1999). Briefly, coherence measures the temporal correlation between two signals through the strength of the consistency of their phase lag as a function of frequency. The result is a coherence spectrum bounded between 0 and 1 for each frequency of interest. A value of 1 indicates a perfect temporal correlation, while 0 indicates no correlation.

For a given time series, *x*(*t*), let the auto spectrum be represented as

$$P_{xx}(f)=\frac{1}{L}\sum_{i=1}^{L}X_{i}(f)\cdot X_{i}^{*}(f)$$

where *X*_*i*_(*f*) represents the Fourier transform of the signal at segment *i* of *L*, and ^*^ indicates the complex conjugate. A similar spectrum exists for the signal *y*(*t*), represented as *P*_*yy*_(*f*). The cross spectrum between the signals *x*(*t*) and *y*(*t*) is represented as

$$P_{xy}\left(f\right)=\frac{1}{L}\sum_{i=1}^{L}X_{i}\left(f\right)\cdot Y_{i}^{*}\left(f\right).$$

Coherence is calculated by normalizing the square of the cross-spectral density between signals *x*(*t*) and *y*(*t*) by the product of their individual auto spectral densities (Baker et al., 1997; Nunez et al., 1997) as represented by

$$C_{xy}\left(f\right)=\frac{{\left|P_{xy}\left(f\right)\right|}^{2}}{P_{xx}\left(f\right)\cdot P_{yy}\left(f\right)}$$

Corticomuscular coherence (CMC) refers to the specific case where cortical and muscular signals represent *x*(*t*) and *y*(*t*). CMC was computed for each EEG-EMG electrode pair as well as FDI-APB coherence using FieldTrip, an open-source toolbox in MATLAB for the analysis of EEG and MEG data (Oostenveld et al., 2011). We used discrete prolate spheroidal sequences (DPSS) or Slepian tapers (Slepian, 1978) for the calculation of the auto and cross spectra. The multi-taper method provides several measures of the spectral estimation by multiplying the data series by a series of orthogonal tapers prior to calculating the Fourier transform (Pesaran, 2008). Three tapers were used in our analysis, providing a spectral bandwidth of ±5 Hz (Maris et al., 2007; Schoffelen et al., 2011).

### Selection of EEG electrodes

Although CMC can be calculated between each muscle and every scalp electrode, we limited our selection to EEG electrodes that showed high FDI-EEG coherence during the dowel-low task. Similar low-force isometric precision task have been previously used in literature for CMC analysis (Baker et al., 1997; Kilner et al., 2000; Fisher et al., 2002; Riddle and Baker, 2006; Chen et al., 2013). Raw coherence values were first normalized by conversion to standard Z-scores using the following formula

$$Z=\frac{\text{arctanh}\left(\sqrt{\text{C}}\right)}{\sqrt{1/2N}}$$

Where N is the total number of tapers used in the calculation of coherence (C) (Baker et al., 2003; Laine et al., 2013, 2014). The electrode locations selected for further analysis were those at which the average *Z*-score exceeded a Bonferroni-corrected 99% confidence level (*Z* = 3.6). The correction accounts for the total number of EEG channels.

### Linear mixed-effects model

A linear mixed-effect model provides a method of describing a relationship for a measurable quantity as a function of the sum of weighted independent variables (Winter, 2013). We investigated the effects of task condition on beta coherence using a linear mixed-effect model with the following format:

$$CMC_{\beta }=\beta_{0}+\beta_{1}\cdot Condition+\beta_{2}\cdot \left(1|Participant\right)+\epsilon$$

where *CMC*_β_ is the average beta-range coherence of an epoch, *Condition* (high and low force for both the spring and dowel) is the fixed-effect term, *Participant* is the random-effect term, β_*n*_ terms are the coefficients for the independent variables, and ϵ is the error. The random-effects term was inserted to account for subject variability since several measurements were taken for each condition. Models were generated to estimate *CMC*_β_ for the FDI and APB well as beta intermuscular FDI-APB coherence.

## Results

### Baseline corticomuscular coherence

**Figure 2:**
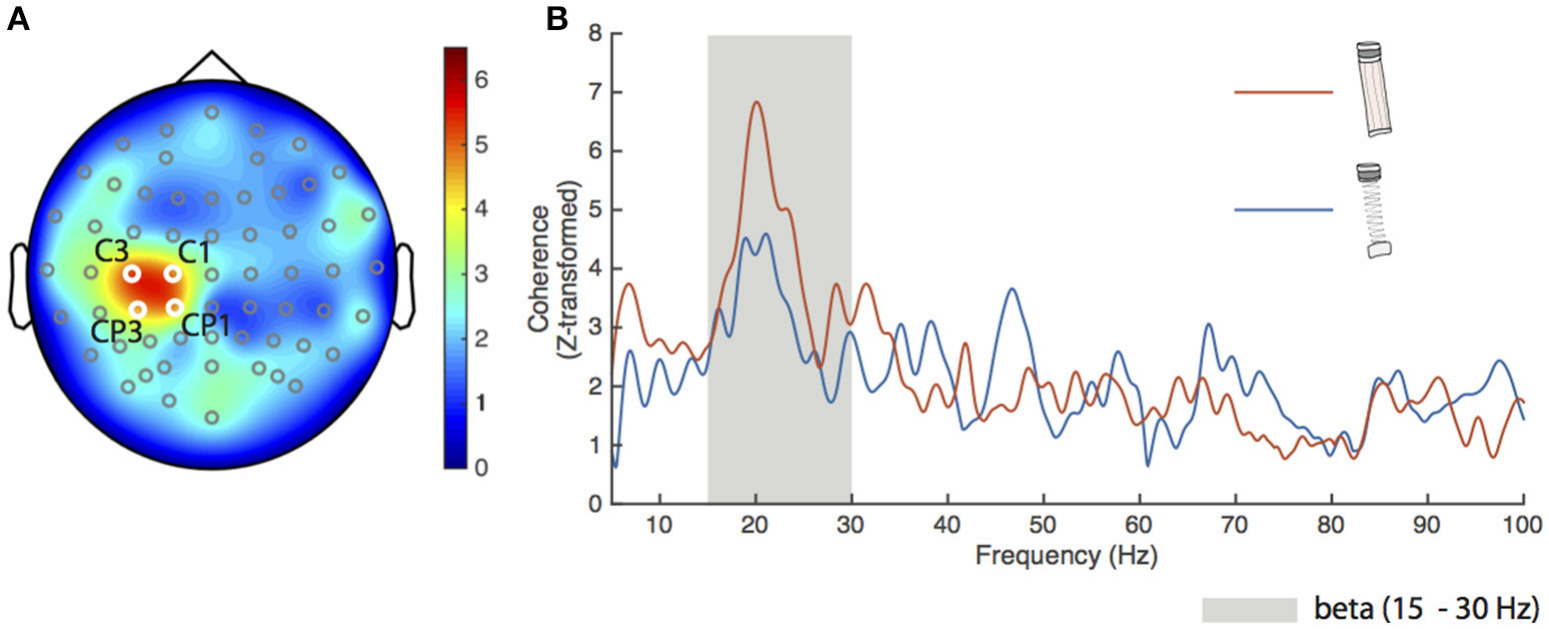
**Results for the dowel-low task. (A)** Grand average Z-transformed FDI-EEG coherence head map for the dowel-low task with all electrode locations marked. The four electrodes used for statistical analyses were C1, C3, CP1, and CP3 (labeled) with respective Z-transformed coherence values of 4.81, 5.26, 4.66, and 4.54. The cluster of electrodes appears over the left sensorimotor cortex. **(B)** Average coherence spectra of the four electrodes shown in **(A)** for the spring-high (blue trace) and dowel-high (orange trace) tasks. The beta frequency band (15–30 Hz) is shown as the gray shaded area. Peak coherence of the average for the spring-low condition was 4.59 at 21.05 Hz and for the dowel-high condition, 6.84 at 20.1 Hz.

To confirm the spatial sensitivity and validity of our analysis procedures, we calculated FDI-EEG coherence for the dowel-low condition. Figure 2A shows a head map of the average Z-transformed corticomuscular coherence between the FDI and all EEG channels. The locations of the four electrodes that exceeded our significance threshold (described previously) are shown in white over the left primary motor cortex and labeled C3, C1, CP3, and CP1. The spatial localization and magnitude shown over the left sensorimotor cortex coincides with previous literature findings during low force production (Witte et al., 2007; Chakarov et al., 2009; Piitulainen et al., 2013).

Cortical drive to the FDI during the dowel-high and spring-high conditions was directly compared. Figure 2B shows the average Z-transformed coherence spectra (5–100 Hz) of the four significant electrodes for the dowel-high (orange trace) and spring-high (blue trace) conditions. The peak coherence for both conditions appeared in the beta frequency range (gray shaded area in Figure 2B). The peak Z-transformed coherence during the dowel-high condition was 6.84 at 20.1 Hz. Despite matched force levels, however, the peak coherence during the spring-high condition decreased to 4.59 at 21.1 Hz.

### Linear mixed-effect model

**Figure 3:**
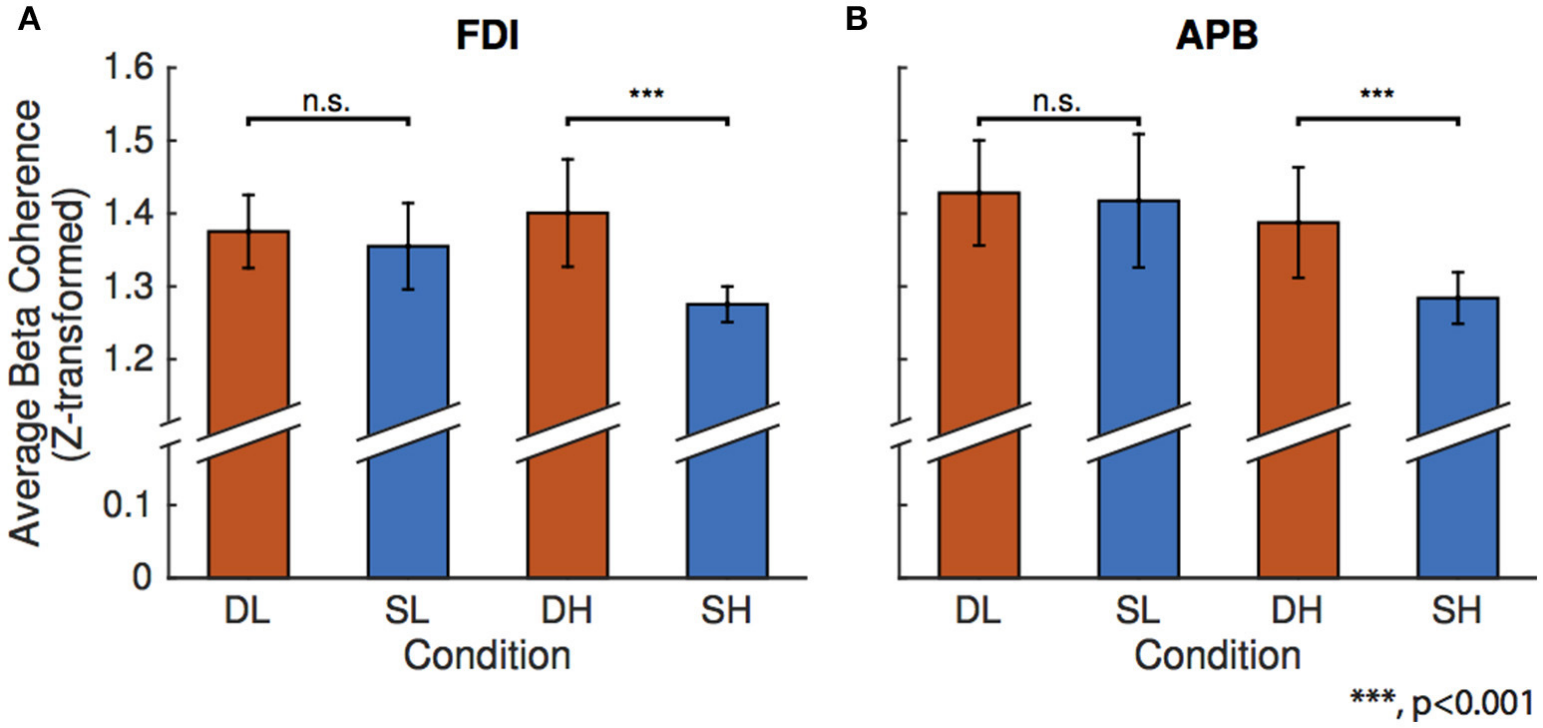
**Results of the linear mixed-effects model for the four conditions: dowel-low (DL), spring-low (SL), dowel-high (DH), and spring-high (SH)**. The model was constructed to predict mean beta range coherence using condition as the fixed-effect with corrections for the random effects of participant. In each bar graph, the mean beta range CMC is shown on the vertical axis and condition is on the horizontal axis. Bars representing one standard error are shown for each condition and significance above the matched force level column pairs represent the statistical difference in the linear mixed-effect coefficients as determined using an *F*-test. **(A)** Linear mixed-effect found no differences in FDI-EEG beta coherence for the low forces, but found a significant difference between the higher force compression conditions. **(B)** Linear mixed-effect model results for APB-EEG coherence show the same statistical results for low and high conditions as in FDI-EEG.

Figure 3 shows the results of the linear mixed-effects model which tests the effects of condition on beta FDI-EEG (Figure 3A) and APB-EEG (Figure 3B) beta-range coherence. Using an F-test, we compared the difference in effect of spring-low and dowel-low model coefficients on beta CMC. We found no significant difference in effect of low force coefficients on either FDI-EEG or APB-EEG beta coherence. It is important to note that, in these low force conditions, both objects remain in the stable domain as the spring has not been compressed enough to exhibit instability.

A similar comparison of the difference in the linear mixed-effect coefficients for the spring-high and dowel-high conditions revealed a significance difference in effect on both FDI-EEG (*p* = 6.8459e-08) and APB-EEG (*p* = 1.6889e-05) beta-range coherence. Overall, CMC was reduced in the unstable task (spring-high condition) compared to the stable task (dowel-high condition) with matched force levels. During the high force compression conditions, the dowel remains in the stable domain, but the spring has been compressed to the point of instability.

### EMG-EMG coherence

**Figure 4:**
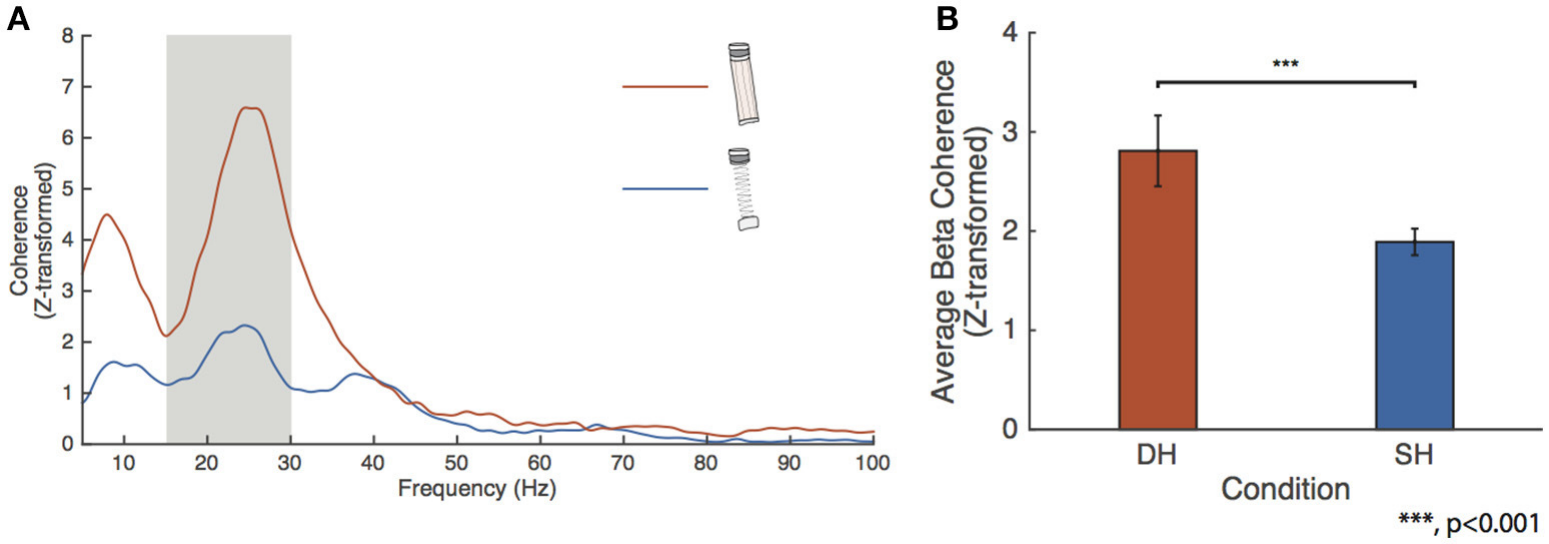
**FDI-APB intermuscular coherence. (A)** Grand average EMG-EMG coherence calculated between the FDI and APB for the spring-high (SH, blue trace) and dowel-high (DH, orange trace) tasks. Peak coherence was 6.59 at 24.63 Hz for the dowel-high condition and 2.33 at 24.44 Hz for the spring-high task. **(B)** Linear mixed-effect model results for the effect of condition on FDI-APB coherence. The effect of dowel-high on beta coherence was significantly higher than for the spring-high condition (*p* < 0.001).

We tested for changes in FDI-APB beta coherence across the higher force conditions. Figure 4A shows the FDI-APB intermuscular coherence for the spring and dowel objects. The dowel-high condition showed a peak beta range Z-transformed coherence of 6.59 at 24.6 Hz. This peak value was significantly higher than for that of the spring-high condition, which had a peak value of 2.33 at 24.4 Hz. Figure 4B depicts the results of the linear mixed-effect model showing that the effects of the dowel was significantly higher than for the spring at the high force level (*p* = 4.9415e-10).

## Discussion

In this study, we have examined the functional meaning of beta-band corticomotor drive. Specifically, we tested the hypothesis that beta-band cortical drive reflects the “binding” of motor neurons into functional units for synergistic cortical control, and thus should depend upon the type/degree of muscle coordination required by a task. Our findings provide evidence that beta-band corticomotor drive is inherently a reflection of intermuscular “binding” rather than steady isometric force production.

It is known that synergistically-activated muscles share beta-band cortical drive, as measured by intermuscular EMG coherence (Kilner et al., 1999; Boonstra and Breakspear, 2012; Boonstra, 2013). It has been suggested that this phenomenon reflects the “binding” of these muscles into a functional unit (or synergy) at the cortical level (Gray, 1994; Santello, 2014). As such, we predicted that beta-band CMC should emerge more prominently when the task of the motor cortex can be reduced to scaling the activation of a functional group of motor neurons (Valero-Cuevas, 2000). The idea that cortical output should reflect the dimensionality of a task is in line with the work of Rathelot and Strick (2006, 2009) who proposed that the motor cortex in humans and some higher primates may have evolved specific pathways (an “old” and “new” M1) to allow optional and flexible utilization of muscle synergies or “motor primitives.”

In this study we have used compression of a slender spring to obligate time-varying fingertip force adjustment while minimizing physical movement and changes in net force (Valero-Cuevas et al., 2003; Dayanidhi et al., 2013a,b; Lawrence et al., 2014). This paradigm has been shown in an fMRI study to increase the engagement diverse brain networks (Mosier et al., 2011) beyond what is required for less-demanding actions. Succeeding at the unstable task (spring-high) requires a level of independent dynamic control of the muscles of the thumb and index finger to dynamically regulate fingertip force vectors to stabilize the spring (Johanson et al., 2001; Venkadesan et al., 2007), which is not the case for isometric tasks like when a wooden dowel is pinched (Valero-Cuevas, 2000). Accordingly, our data showed a general reduction of intermuscular coherence between the EMG signals recorded from the APB and FDI muscles when the spring was compressed at a level that induced instability. The reduction of beta-band (15–30 Hz) coherence between muscles can be interpreted as stemming from an “unbinding” of these two muscles at the level of the cortex. Changes in force alone would not explain our results, since little change in IMC was observed in the dowel-low vs. dowel high condition. Further, increased spring compression force is associated with increased instability, but not changes in spring compliance. Our results almost certainly reflect a neural reaction to the demands of controlling instabilities.

Similar to our findings for IMC, CMC in the beta-band was markedly reduced for the FDI and ABP muscles when the spring became unstable in the spring-high condition. If this phenomenon were simply due to increased noise in the EEG signal (e.g., due to increased cortico-cortical communication during the more difficult task) we would expect that IMC would have been preserved (since the muscles would still share beta-band cortical drive), but this was not the case. Further, a change from static to dynamic force production is known to increase high-frequency (30–50 Hz) CMC as beta-band CMC shifts to higher frequencies (Omlor et al., 2007). We did not observe this type of shift. The simplest interpretation would be that the beta-band cortical drive was fundamentally an intermuscular signal in this task, and that a shift in cortical control strategy occurred when the thumb and index finger muscles required independent control. This interpretation is also in line with evidence from a pilot study in which within-muscle motor unit coherence was reduced in the APB muscle during the same high-force spring compression task (Laine et al., 2015). A relationship between beta-band cortical drive and control strategy also agrees with its dependence on psychosensory aspects of a task (Laine et al., 2014), although typically it is the higher frequencies of cortical drive (30–50 Hz) which show the greatest sensitivity to such features (Brown et al., 1998; Omlor et al., 2007, 2011; Patino et al., 2008; Mehrkanoon et al., 2014).

Recently it has been hypothesized that beta oscillations arise from closed loops from M1 to muscle synergies back to M1 (Aumann and Prut, 2015). As such, during sustained contraction of a muscle, groups of sensorimotor neurons oscillate in synchrony to maintain the current state (Engel and Fries, 2010). Conversely during movement, de-synchrony of the local group disrupts beta rhythms. The results obtained in this study support this closed-loop hypothesis given in the fact that during the stable conditions (i.e., spring-low, dowel-low and dowel-high), synergistic muscle activations were necessary for producing the target forces. However, in the spring-high condition, it was necessary to disrupt these synergies despite the fact that a relatively constant net force was maintained.

### Confounds and alternative explanations

Physical differences in the objects compressed are of key importance to this study. Our interpretation is that the primary factor was the decreased stability of the spring when compressed at higher forces, however, small differences finger position, movement, object compliance, etc. could influence coherence measures and must be considered.

In a previous study, Kilner et al. found positive correlations between the magnitude of beta-band CMC and object compliance during a ramp and hold precision pinch task (Kilner et al., 2000). In our study, we did not observe a difference in beta-band CMC between the dowel-low and spring-low conditions, and at high forces, we observed a reduction rather than an increase in beta-band CMC. While the study of Kilner et al. shares many similarities with the work presented here, the compliant object used in our study was free at both ends and had the propensity to buckle and slip out of the hand when compressed at applied forces >2.2 N. In contrast, two fixed levers with programmable compliance were compressed in the study of Kilner et al. Such differences make direct comparison between studies inexact, but it is clear that the higher compliance of the spring relative to the dowel should, if anything, favor beta-band coherence (Kilner et al., 2000), as should higher force relative to lower force (Witte et al., 2007). Accordingly, our observation of decreased beta-band coherence in the spring-high condition appears to be related to a change in neuromuscular control strategy in response to the reduced stability of the spring. If very small movements can influence CMC (Kilner et al., 1999), then it is possible that the underlying reason for this may relate to the fact that movement may not allow muscles to be “bound” in the same way as is possible during static isometric force production. Although the physical positioning of the fingers can also have an influence on coherence (Riddle and Baker, 2006) the final position of the fingers during spring vs. dowel compression did not differ by more than about 1 cm, and we have no reason to believe that this would have been a major factor in the present study. Moreover, prior fMRI work in this same task has shown that the presence of instability has an effect distinct from that of compliance (Mosier et al., 2011).

It is also important to consider potential drawbacks of using EEG and surface EMG signals. For example, surface EMG will not be as sensitive as single motor unit recordings (Keenan et al., 2012) and may be influenced by signal processing techniques such as filtering or rectification (Boonstra and Breakspear, 2012; Farina et al., 2013). Even so, a systematic difference in the sensitivity of EMG signals to beta-band drive across tasks seems an unlikely explanation for our results, especially given the low levels of force required and our use of the most appropriate signal processing methods (i.e., EMG rectification) under these conditions (Farina et al., 2013).

It is relevant that compression of the spring to the point that it becomes unstable is an inherently difficult and demanding task. While there is increasing evidence that M1 activity may be involved with the perception of task goals (Shen and Alexander, 1997; Cisek et al., 2003; Scott, 2003), our suggestion is that the perception of difficulty in motor tasks is secondary to more tangible factors, such as the need for time-critical sensorimotor corrections, and individuated vs. synergistic control of muscles. In one recent study, for example, beta-band CMC was reduced when the degree of bimanual muscle coordination was increased through a visual feedback manipulation (de Vries et al., 2016). The interpretation was that beta-band CMC relates to the control of individual muscles, rather than coordination. The opposite argument could also be made, however, because in that study, errors of force may have been perceived and corrected individually for each hand in order to achieve better overall bimanual coordination. This would be in line with our coordination-based interpretation of beta-band drive to muscles. The same study also found that low-frequency IMC (~10 Hz) across hands was highest when bimanual coordination was highest. Because ~10 Hz CMC is not present in that study nor our own, low-frequency drive to individual muscles cannot be measured or compared concurrently with inter-muscular drive (i.e., IMC). This makes it difficult to attribute changes in IMC to inherently coordination-related aspects of our tasks, rather than other factors which might influence the production of low-frequency neural drive in general. That said, our Figure 4A does visibly show low-frequency IMC reduced along with the beta-band in the spring-high condition, which certainly justifies further investigation of this issue.

Overall, our study tests the notion that beta-band cortical drive essentially reflects the dimensionality of cortical commands, and our results strongly suggest that beta-band CMC should be interpreted carefully, with special attention to muscle coordination and time-critical sensorimotor demands. Our current speculation, supported by the findings of this investigation, is that beta-band CMC reflects the use of a low-dimensional mode of cortical control over groups of muscles, rather than the maintenance of a steady-state force output. While fully understanding the neurophysiology and task-dependence of cortico-motor oscillations requires further study, our results justify such future work and provide a springboard for more focused investigations into the relationship between the physical requirements of a task and neural control strategies necessary to satisfy them. Finally, such insights into the origin and modulation of cortico-motor oscillations would not only clarify fundamental mechanism for sensorimotor control, but perhaps provide well-founded tasks and analyses directly translatable to clinical measures and diagnostic tests (Norton and Gorassini, 2006; Hammond et al., 2007; Pogosyan et al., 2009; Fisher et al., 2012; Ko et al., 2016).

## Author contributions

AR, JK, and FV designed research; AR performed research; AR, CL, and JK analyzed data; AR, CL, JK, and FV wrote the paper.

### Conflict of interest statement

FV holds US Patent No. 6,537,075 on some of the technology used in this study that is commercialized by Neuromuscular Dynamics, LLC. The other authors declare that the research was conducted in the absence of any commercial or financial relationships that could be construed as a potential conflict of interest.
